# Calcium Unresponsive Hypocalcemic Tetany: Gitelman Syndrome with Hypocalcemia

**DOI:** 10.1155/2013/197374

**Published:** 2013-09-19

**Authors:** Madhav Desai, Praveen Kumar Kolla, P. L. Venkata Pakki Reddy

**Affiliations:** Department of Nephrology, Narayana Medical College, Chintareddypalem, Nellore, Andhra Pradesh 524003, India

## Abstract

*Introduction*. Gitelman's syndrome (GS) is autosomal recessive renal tubular disorder characterized by hypokalemia, hypomagnesemia, hypocalciuria, metabolic alkalosis, and hyperreninemic hyperaldosteronism. It is usually associated with normal serum calcium. We report a patient presented with hypocalcemic tetany, and evaluation showed Gitelman's syndrome with hypocalcemia. *Case Report*. A 28-year-old woman presented with cramps of the arms, legs, fatigue, and carpal spasms of one week duration. She has history of similar episodes on and off for the past two years. Her blood pressure was 98/66 mmHg. Chvostek's sign and Trousseau's sign were positive. Evaluation showed hypokalemia, hypocalcemia, hypomagnesemia, metabolic alkalosis, and hypocalciuria. Self-medication, diuretic use, laxative abuse, persistent vomiting, and diarrhoea were ruled out. Urinary prostaglandins and genetic testing could not be done because of nonavailability. To differentiate Gitelman syndrome from Bartter's syndrome (BS), thiazide loading test was done. It showed blunted fractional chloride excretion. GS was confirmed and patient was treated with spironolactone along with magnesium, calcium, and potassium supplementation. Symptomatically, she improved and did not develop episodes of tetany again. *Conclusion*. In tetany patient along with serum calcium measurement, serum magnesium, serum potassium, and arterial blood gases should be measured. Even though hypocalcemia in Gitelman syndrome is rare, it still can occur.

## 1. Introduction

Gitelman syndrome (GS) was first described by Gitelman et al. in 1966 [1]. The prevalence of Gitelman syndrome is 25 per million population. GS is autosomal recessive disorder caused by mutations in the SLC12A3 gene present on the chromosome 16q13 encoding the thiazide-sensitive sodium chloride contrasporter (TSC) at the distal tubule. It is characterized by hypokalemia, hypomagnesemia, hypocalciuria, metabolic alkalosis, and hyperreninemic hyperaldosteronism. In majority of patients of GS serum calcium is normal. We report a patient presented with hypocalcemic tetany, and evaluation showed Gitelman's syndrome with hypocalcemia.

## 2. Case Report

A 28-year-old woman presented with complaints of cramps of the arms, legs, fatigue, and carpal spasms of one-week duration. She has been suffering with the similar complaints on and off for the past two years. Self-medication, diuretic use for prolonged periods, laxative abuse, persistent vomiting, and diarrhoea were ruled out. Patient did not have symptoms of polyuria and polydipsia. No family history of similar symptoms is reported. No growth retardation and bone deformities are noted. Her blood pressure was 98/66 mm Hg with no postural hypotension. Clinically she was euvolemic. Chvostek's sign and Trousseau's sign were positive. Investigations are shown in Table 1. Arterial blood gases (ABG) showed hypochloremic metabolic alkalosis [pH 7.51 (7.35–7.45), HCO_3_^−^ 33.5 (22–30), P_CO_2__ 42 (32–45), and chloride 86 (102–109) mEq/L]. The following investigations were within normal limits: complete blood picture (CBP), fasting plasma glucose, serum creatinine, liver function test, thyroid profile, rheumatoid factor, and anti-nuclear antibodies (ANA). Ultrasound imaging of kidneys was normal with no medullary nephrocalcinosis. Urine pH was 6.7 and specific gravity was 1.018.

 Urinary prostaglandins and molecular genetic studies were not performed owing to lack of facilities. The thiazide test (hydrochlorthiazide) was performed as described by Colussi et al. [2]. After an overnight fast, the patient was kept recumbent for 4 hours and was given water (10 mL/kg body weight) to facilitate spontaneous voiding. After two 30 minutes basal clearances (mean of two values was taken), 50 mg of hydrochlorthiazide was administered orally, and six additional 30-minute clearances were performed. Serum chloride (Cl) and creatinine were measured at 1 hour and 4 hours. Urine was collected every 30 minutes by spontaneous voiding and analyzed for chloride and creatinine. After written consent, a healthy person was taken as control. The chloriuretic effect was higher than the natriuretic effect due to greater sodium (Na) than Cl reabsorption in tubular segments past the site of the diuretic action; hence, Cl data are usually preferred over Na in diuretic based clearances. The diuretic effect was measured by choosing maximal increase of urine electrolyte excretion over mean basal levels (i.e., maximal excretion at any time minus the mean of the two basal clearances) as the test result. Fractional excretion of chloride is as follows:

(1) FeCl %=100    ×[Urine  chloride (Ucl⁡)×Plasma  creatinine (Pcr)][Plasma chloride  (Pcl⁡)×Urine creatinine  (Ucr)].

Results of this test were shown in Table 2.

Hypocalcemia was treated initially with 10 mL of 10% wt/vol (1 gram of calcium gluconate equivalent to 90 mg of elemental calcium) calcium gluconate diluted in 50 mL of 5% dextrose is given intravenously over a period of 10 minutes. It was followed by slow infusion at a rate of 50 mg of elemental calcium per hour (1 mg elemental calcium/kg/hour). Oral calcium supplementation was started at a dose of 1500 mg of elemental calcium daily. Hypomagnesemia was treated with intravenous magnesium sulphate administered at a rate of 64 mEq over the first 24 hours and then 32 mEq daily for the next 2 days followed by oral magnesium salts. Hypokalemia was treated with oral potassium chloride solution 40 to 100 meq/L per day, divided into three doses. Doses of intravenous and oral supplementations of calcium, potassium, and magnesium were adjusted according symptoms and serum levels electrolytes. Spironolactone 200 mg in two divided doses was also added. The patient's symptoms had improved partially. Serum potassium and magnesium levels were corrected to 3.2 mEq/L and 1.7 mEq/L, respectively. Serum calcium was corrected to 8.6 mg/dL. She did not develop further episodes of tetany.

## 3. Discussion

When patient presented with features suggestive of tetany, the initial investigations showed hypocalcemia. Despite treatment with intravenous and oral calcium, the tetany did not subside. Further evaluation showed hypomagnesemia, hypokalemia, and metabolic alkalosis. These investigations suggested the possibility of two causes either Bartter's syndrome (BS) or Gitelman syndrome. Hypomagnesemia cannot differentiate between BS from GS because 20–30% of BS patients can have mild hypomagnesaemia [7]. Hypomagnesemia in Bartter's syndrome is due to loss of thick ascending loop of henle (TAL) transepithelial potential difference that drives paracellular divalent cation reabsorption. In GS hypomagnesaemia is severe and seen in 100% patients. Muscle cramps and chondrocalcinosis are commonly seen in GS and not seen in BS. Hypocalciuria (calcium and creatinine ratio less than 0.2) in this patient suggested the GS [8]. As molecular genetic tests were not available, thiazide loading test was done. The test showed blunted chloriuretic response with thiazide diuretic in this patient when compared with the control (ΔFe_cl⁡_ 1.8 in patient and 3.7 in control). When compared with historic control also, the diuretic response is blunted (ΔFe_cl⁡_ 1.8 in this patient, 2.3 (3.58 ± 1.00) in historic control [2]). 

The cause of hypocalcemia, in spite of hypocalciuria in GS, is due to hypomagnesaemia induced (a) impaired synthesis, secretion of PTH [9] (b) end organ resistance to PTH and vitamin D [10]. Usually in GS serum calcium levels are normal and tetany is because of hypomagnesemia. But this patient has GS with both hypocalcemia and hypomagnesemia.

Our patient data was compared with the other reported cases in the literature (Table 3).

 Another differential diagnosis for hypomagnesemia with hypocalcemia includes hypomagnesemia with secondary hypocalcemia (HSH). It manifests in the newborn period and is characterized by very low serum magnesium and low calcium concentrations with normal serum potassium levels. 

## 4. Conclusion

In tetany patient along with serum calcium measurement, serum magnesium and arterial blood gases should be measured. Even though hypocalcemia in Gitelman syndrome is rare, it still can occur.

## Figures and Tables

**Table 1:** Investigations.

Parameter	Value	Reference range and units
Serum sodium	138	135–145 mEq/L
Serum potassium	**2.5**	3.5–5 mEq/L
Serum calcium	**7.3**	8.7–10.2 mg/dL
Serum phosphorus (inorganic)	3.0	2.5–4.3 mg/dL
Serum magnesium	**1.0**	1.5–2.3 mg/dL
Intact parathyroid hormone	35	15–65 pg/mL
25 OH Vitamin D	52.1	23–113 nmol/L
Plasma renin	**7.2**	0.2–1.6 ng/mL/hour
Serum aldosterone	**172**	30–160 pg/mL
24 hours urine		
(i) volume	2000	mL/day
(ii) sodium	229	40–220 mmol/day
(iii) potassium	146	25–125 mmol/day
(iv) magnesium	168	70–130 mg/day
(v) calcium	**205**	420–560 mg/day
(vi) phosphorus	482	400–1300 mg/day
(vii) calcium/creatinine	**0.13**	<0.2 in GS, >0.2 in BS
(viii) protein	120	30–150 mg/day

**Table 2:** 

	FE_cl (basal%)_	FE_cl (Max-post thiazide%)_	ΔFe_cl_
Patient	1.9	3.7	1.8
Control (healthy person)	3.1	7.8	3.7

FE_cl (basal%)_: basal fractional excretion of chloride; FE_cl (Max-post  thiazide%)_: post thiazide maximal fractional excretion of chloride; ΔFe_cl⁡_: maximal “increase” of chloride after thiazide.

**Table 3:** GS with hypocalcemia.

	Present study	Nakamura et al. [3]	Ran et al. [4]	Yeum et al. [5]	Al-Ali et al. [6]
Country	India	Japan	China	Korea	Kuwait
Patient age (years)	28	18	63	16	35
Sex	Female	Male	Female	Female	Female
Presentation	Tetany	Muscle weakness and osteopenia	Recurrent hypokalemia paralysis	Muscle weakness	Muscle weakness and carpal spasm
Confirmation	Diuretic test (blunted chloriuretic response with thiazide)	Genetic analysis	Diuretic test	Diuretic test	Hypocalciuria
